# Effectiveness of a group intervention to reduce the psychological distress of healthcare staff: a pre-post quasi-experimental evaluation

**DOI:** 10.1186/s12913-021-06413-4

**Published:** 2021-04-27

**Authors:** Jeremy Dawson, Imelda McCarthy, Cath Taylor, Kristin Hildenbrand, Mary Leamy, Ellie Reynolds, Jill Maben

**Affiliations:** 1 Sheffield University Management School, University of Sheffield, Conduit Road, Sheffield, S10 1FL UK; 2 Aston University, Birmingham, UK; 3 University of Surrey, Guildford, UK; 4 King’s College, London, UK

**Keywords:** Schwartz rounds, Intervention study, Psychological distress, Engagement

## Abstract

**Background:**

Work stress and compassion fatigue are prevalent among healthcare staff and their negative effects on staff well-being and patient care are well-known. This paper reports on the implementation and evaluation of Schwartz Rounds® (Rounds) in UK healthcare organizations, predominantly part of the National Health Service (NHS). Rounds are one-hour, typically monthly, multidisciplinary forums during which clinical and nonclinical healthcare staff discuss the emotional and social demands of delivering patient care. The purpose of this research was to evaluate the effectiveness of Rounds attendance on the psychological distress, work engagement, compassion and self-reflection of healthcare staff.

**Methods:**

We used a pre-post control design to assess the effect of Rounds attendance across 10 UK healthcare organizations. This design was most appropriate given the voluntary nature of Rounds and ensured the study had ecological validity. Self-reported data were collected from attenders and non-attenders at baseline and at eight-months follow-up. The outcomes were psychological distress, work engagement, compassion and self-reflection.

**Results:**

During the 8 months’ study duration, regular attenders (*N* = 51) attended Rounds on average 4 times (2–8). Attenders showed a significantly greater decrease in psychological distress (as measured with the General Health Questionnaire (GHQ)) than non-attenders (*N* = 233; odds ratio of 0.197; 95% confidence interval (0.047–0.823)). However, Rounds attendance had no significant effect on work engagement, compassion and self-reflection.

**Conclusions:**

Rounds attendance was linked to a 19% reduction in psychological distress adjusting for covariates. As an organization-wide intervention, Rounds thus constitute an effective, relatively low-cost intervention to assist staff in dealing with the demands of their work and to improve their well-being.

**Supplementary Information:**

The online version contains supplementary material available at 10.1186/s12913-021-06413-4.

## Background

The work of healthcare staff is characterized by various ethical, moral, social and emotional challenges and demands [1, 2], such as caring for a large number of patients, interacting with their families, or dealing with patients’ deaths [1, 3]. Such demanding work comes at a cost [4], as reflected in rising levels of burnout and compassion fatigue and sadly, psychological morbidity [5–8]. While highly problematic in itself, poor staff health also affects important patient outcomes, such as quality of patient care, patient safety and patient mortality [9–11]. Different interventions that aim at improving staff well-being and compassion have been proposed and implemented. The 2013 Francis Report [12], which examined high profile failings in care, points towards Schwartz Rounds® (Rounds) as a method to improve staff compassion, well-being and patient care. Key benefits of Rounds over comparative interventions (e.g., resilience training and after action reviews) include that Rounds do not require verbal contribution on part of attenders and are open to clinical and non-clinical staff [13]. The latter point is particularly relevant in the context of well-being and burnout as ‘all staff’ are exposed to the various challenges inherent in patient care. This study therefore focuses on exploring the effectiveness of Rounds in improving well-being of staff.

Rounds are organization-wide, multidisciplinary forums that have primarily been implemented in healthcare organizations (over 480 in the US and over 220 in the UK and Ireland [14], including acute, community and mental health hospitals and hospices [15]. Rounds are run consistently, with high fidelity [16] as set out in the contract between the Schwartz Center for Compassionate Care (USA) and the Point of Care Foundation (UK). As opposed to other interventions, Rounds are voluntarily attended by clinical and nonclinical staff [13, 14]. Rounds can be classed as a group intervention [13, 14], with attendance (staff can attend as few, or many as desired, or none) usually between 30 and 100+ members of staff. They are typically held monthly during lunchtime with lunch provided, last 1 hour, and begin with a brief, multi-disciplinary panel presentation of a patient case or a set of similar experiences relating to a theme or Rounds title [14]. During the presentation, panel members share their experiences of work issues or of caring for patients, focusing on their social, psychological and/or emotional impact. Subsequently, the audience participates in a discussion, co-facilitated by a clinical lead, who is a senior clinician, and a facilitator trained in leading Rounds. This discussion involves an open exchange and reflection on similar work or caring experiences [13]. Thus, in contrast to traditional medical rounds, Rounds provide healthcare staff with the space to reflect upon the human dimensions of their work, enabling them to gain insight into their own and others’ responses, contributing to recognition or development of their empathy and compassion. Rounds thus support staff to better cope with the ethically, morally and emotionally difficult aspects of their jobs, as opposed to focusing on the clinical aspects of care, problem solving or developing action plans [13, 14].

Despite the widespread implementation of Rounds, the evidence base for their effectiveness is limited [13]. Specifically, the majority of evaluations explored attenders’ experience of Rounds via post-intervention evaluation sheets or interviews. These studies showed that Rounds were generally perceived as beneficial and attenders reported increased reflection and compassion, ability to walk in another’s shoes, and empathy [17–19]. They also highlighted that Rounds reduce healthcare staff’s work stress [19–21] and that improving staff’s well-being was the primary reason why healthcare organizations implemented Rounds in the UK [15, 20–22]. While these studies offer initial insights, the use of only single time, post-intervention assessments, susceptible to mood effects and unable to capture change in outcomes, represents weak evidence for the effectiveness of Rounds. We are aware of a few evaluation studies that address these concerns through evaluating Rounds with data collected before and after Rounds attendance [19, 23, 24]. However, as these studies did not include a control group, it is not possible to attribute the observed changes to Rounds attendance. In sum, as existing evaluations relied on retrospective accounts, did not include baseline measurements, and did not include a control group [22, 23], the quality of the existing evidence base regarding the effectiveness of Rounds is relatively low [13].

This project thus aimed to evaluate the effectiveness of Rounds in the UK healthcare sector with a pre-post control design. Using a superior research design enabled us to address the mentioned limitations of previous research and yield more robust and reliable evidence regarding the effect of Rounds on employee outcomes. Our choice of outcome variables was informed by the Job Demands-Resources model (JD-R) [25], a well-established occupational stress model that has been extensively leveraged to study employee health and motivation. Specifically, according to the health impairment route [26], job demands, aspects of a job that require sustained physical, emotional, or cognitive effort [25], such as working under time pressure or dealing with difficult patient cases, contribute to employees’ psychological distress. This negative process can be offset by job resources, defined as those aspects of a job that stimulate personal growth and development [25] or personal resources, defined as those personal traits and energies that enable individuals to control and impact their environment [27]. According to the motivational route of the JD-R model [26], both types of resources increase employees’ motivation and work engagement. Schwartz Rounds were designed to demonstrate that compassion can be taught and to improve connection and compassionate care for patients, which is thought to be partly achieved through role modelling, such as senior clinicians revealing their vulnerability and sharing their experiences [16]. Both compassion and self-reflection represent personal resources and we thus expected healthcare staff to experience an increase in these personal resources following Rounds attendance. Consequently, we hypothesized that Rounds attendance would lead to a reduction in healthcare staff’s psychological distress and an increase in their work engagement, compassion and self-reflection. In the following sections, we describe and discuss a pre-post quasi experimental evaluation of Rounds in 10 UK healthcare organizations that tested these hypotheses.

## Methods

### Research design

This survey study was conducted as part of a wider evaluation of Rounds in England. A description of the wider study can be found in the full report to the funding body [28]. Ethical approval was granted by the National Research Ethics Service Committee London-South East (REC reference 15/LO/0053). The design of the study was a two-armed, longitudinal (pre-post) control design, with the two arms being regular attenders of Rounds and non-attenders of Rounds. Participants qualified as ‘regular attenders’ if they had attended 50% of the Rounds their respective healthcare organization offered during the eight-month evaluation period, but had not attended Rounds before that; non-attenders had not attended any Rounds at all. The pre-post control design was chosen over a randomized control trial to increase the ecological validity of the study as Rounds attendance is always voluntary and our intervention design thus mirrored the unpredictable and uncontrollable pattern of Rounds exposure [29]. We predicted attendance at Rounds would reduce participants’ psychological distress; withholding this intervention from participants (as would be required with a randomized control group) thus seemed inappropriate as it would cause an ethical dilemma [30].

### Study population and sample

The study was conducted in ten healthcare organizations in the UK, of which eight were hospitals, one provided mental health services (both inpatient and community), and one was a hospice. Other than the hospice, all organizations were part of the NHS. Of the 10 organizations, four had been running Rounds for between one and 6 years before the study; the other six were new implementers of Rounds. Baseline data collection began at each site between February and September 2015, with the baseline data collection period lasting typically around 4 months at each site, and follow-up data collected 8 months after the baseline data.

The desired sample size was calculated prior to the study. To achieve 80% power of detecting a small-to-moderate effect (d = 0.40), with a two-tailed alpha level of .05 and allowing for a modest clustering effect (an inter-cluster correlation of 0.02), a sample of 114 participants per arm was required. We assumed a 50% response rate for the follow-up questionnaire of attenders, that around 50% of these would fulfil our attendance criteria of 50% of Rounds offered, and that 10% of non-attenders would respond to both questionnaires. This meant we had an overall target sample of 456 attenders and 1140 non-attenders.

To be included in the study, participants must not have attended any Rounds before the baseline survey, and must have been an employee of the healthcare organization hosting Rounds. They also needed to have completed questionnaires at both baseline and follow-up. To meet the criterion of being a regular attender, they should have attended at least 50% of the Rounds available at their site. Therefore, participants from the control arm, who met the regular attender inclusion criterion, were also included as attenders in the analysis. To qualify as a non-attender, the only additional inclusion criterion was that they had never attended any Rounds by the time of the follow-up survey.

The two arms were recruited in different ways. To recruit attenders, up to four members of the research team attended up to four successive Rounds at each site, arriving at least 30 min prior to the Rounds so that they could approach attenders as they arrived. Eligible participants who had not attended a Round previously were invited to participate in the study, which involved completing a short questionnaire before they attended their first Round (baseline) and 8 months thereafter (follow-up). The baseline questionnaire (a copy of which can be found in the supplementary material) was usually completed before the Round began, though sometimes participants completed these during or shortly after the first Round. Participants’ contact information was collected via a separate cover sheet, in order to be able to send follow-up questionnaires and link them with baseline questionnaires. Eight months later, these follow-up questionnaires were distributed electronically unless the participant had expressed a preference for a paper-based questionnaire. A gap of 8 months was chosen for pragmatic reasons: it was short enough to allow the study to be conducted in a timescale that would provide useful results reasonably quickly, but it was also long enough so that 50% attendance would result in attending four Rounds, if they were available monthly – this “dose” suggested by previous research as being sufficient to have a positive impact [23]. (Most organisations ran Rounds monthly, but not always on the same physical site; the ability of staff to travel between sites varied.)

For the control arm, a random sample of 250 staff per organization (or all staff if the organization was smaller than this) was selected from a staff list, and contacted by email with an invitation to complete an electronic version of the baseline questionnaire. We deliberately over-recruited compared with the target sample size because we did not know what proportion of those sampled would have attended Rounds previously. If respondents had ever attended a Round, they were automatically excluded from the study before actually completing the baseline questionnaire. If respondents had never attended a Round, they received the follow-up questionnaire 8 months later.

### Outcome measures

Twelve cognitive interviews and a pilot study in two organizations were conducted in order to test the applicability of the measures and methods of data collection [28]. All outcomes were measured at both baseline and eight-months follow-up.

Psychological distress was measured with the 12-item version of the General Health Questionnaire (GHQ) [31]. We used a validated scoring method [32], which determined whether participants were “cases” or not – that is, they were considered to be in sufficiently poor psychological health that they would benefit from professional intervention. They were classified as a “case” if they scored in the highest two response categories on at least four of the twelve items. Work engagement was measured with the three-item “motivation” section of the NHS staff survey (ww.nhsstaffsurveys.com), which is a brief version of the Utrecht Work Engagement Scale [33]. Participants responded on a five-point Likert scale (i.e., 1 = never, 5 = always).

Self-reflection was measured with a six-item self-reflection subscale taken from a scale on self-reflection and insight [34]. Participants responded on a seven-point Likert scale (1 = strongly disagree,7 = strongly agree). Compassion (in a general sense, including towards patients) was measured with a five-item version [35] of the Santa Clara Brief Compassion Scale [36]. Participants responded on a seven-point Likert scale (1 = not at all true of me, 7 = very true of me).

#### Covariates

Respondents indicated their occupational group (medical/dental, nursing, allied health professionals or other), grade (NHS bands 1–5, band 6, band 7, bands 8/9, or other; typically bands 1–5 represent relatively junior positions, bands 6 and 7 represent core clinical groups other than medical staff, and bands 8 and 9 represent those with middle and senior managerial responsibility), gender, age, length of service, part-time or full-time status, and extent of contact with patients (regular, occasional or none) at baseline. The GHQ-12 is under license, and a license was obtained for specific use in this study. None of the other measures is under license.

### Statistical analysis

Multilevel (respondents within sites) analysis was used to estimate the effect of attendance on the outcomes. In particular, as psychological distress was measured with a binary score, multilevel logistic regression was used, controlling for baseline psychological distress and the covariates. For the other outcomes, a multilevel ANCOVA was used, controlling for baseline scores and the covariates. For the primary analysis listwise deletion of any missing demographic data was used, but sensitivity analysis conducted using Full Information Maximum Likelihood confirmed the findings. Participants who did not include data on follow-up measures, or the number of Rounds attended, were excluded from the analysis.

### Patient and public involvement

Patients were involved throughout the wider study, with two patient representatives as part of the project steering committee. This ensured oversight and input into of all aspects of the study, including development of the research questions, study design, and choice of outcome measures for all parts of the study, including the survey sub-study covered by this article. Patients were included in this way, even though the study did not directly involve patients as participants, to ensure that the relevance to patient care and quality was never lost.

## Results

### Participants

**Fig. 1:**
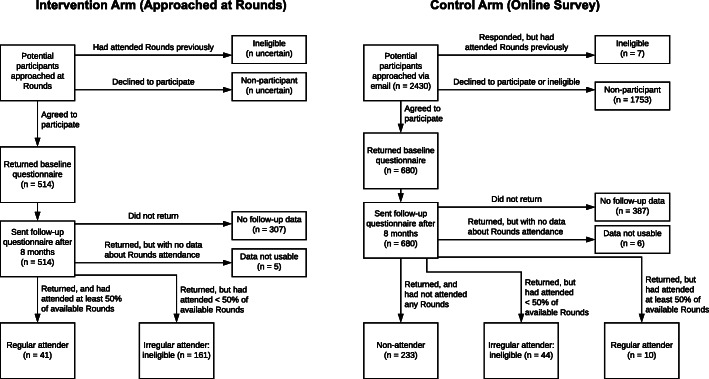
Flow Diagram of Participants

There were a total of 1194 respondents at baseline, of whom 500 completed the follow-up questionnaire. Of these, 51 met the criteria for being included as a regular attender. The majority of these participants were audience members at Rounds, who were able to contribute as much or as little as they wanted during discussions, although 15 participants were panel members at one of the Rounds they attended. Another 233 had not attended Rounds at all and were thus included as non-attenders. The remaining respondents had either attended fewer than 50% of available Rounds (irregular attenders), or did not provide sufficient information to determine the number of Rounds attended, and were therefore excluded from the analysis. We include a flow diagram showing how participants in both arms were included and classified (Fig. 1). It was not possible to calculate a response rate of attenders, as we were not able to record the number of employees approached in total. In the control arm, the baseline online questionnaire achieved a response rate of 28%; however, an unknown number of potential respondents might have excluded themselves and not completed the baseline survey due to prior Rounds attendance. The follow-up questionnaire achieved response rates of 40% in the intervention arm and 43% in the control arm. Of the regular attenders, the mean number of Rounds attended was 4.0, with a range from 2 to 8 (interquartile range 3–5).

### Baseline characteristics

Table 1 shows the baseline characteristics of the two groups, regular attenders and non-attenders. Although they are similar in many variables, some differences are notable: in particular, non-attenders are more likely to come from non-clinical groups, more junior grades, and have less contact with patients. Table 2 shows the data for the outcomes at both baseline and follow-up by arm. There is a clear difference in baseline GHQ between the two arms, with attenders having lower rates of psychological distress. Attenders also had higher baseline scores for work engagement, and self-reflection, and slightly higher for compassion.

**Table 1:** Descriptive statistics for baseline characteristics

Variable (number of cases with missing data)	Regular attenders (*N* = 51)	Non-attenders (*N* = 223)
Gender: female (11)	88%	79%
Occupational group: medical/dental	14%	6%
Occupational group: nursing	44%	38%
Occupational group: allied health professionals	32%	22%
Occupational group: other (14)	10%	35%
Working hours: full time (19)	91%	81%
Grade: bands 1–5	23%	49%
Grade: band 6	20%	24%
Grade: band 7	37%	15%
Grade: bands 8/9	14%	10%
Grade: other (37)	6%	3%
Regular patient contact	82%	72%
Occasional patient contact	12%	11%
No patient contact (34)	6%	17%
Age^a^ (11)	3.88 (1.08)	3.93 (1.05)
Length of service^b^ (18)	4.00 (1.93)	4.08 (1.63)

^a^ Coded as 1 = 16–20, 2 = 21–30, 3 = 31–40, 4 = 41–50, 5 = 51–65, 6 = 66+

^b^ Coded as 1 = up to a year, 2 = 1–2 years, 3 = 3–5 years, 4 = 6–10 years, 5 = 11–15 years, 6 = over 1 years

**Table 2:** Summary statistics for outcomes at baseline and follow-up

	Regular attenders		Non-attenders	
	Baseline	Follow-up	Baseline	Follow-up
Psychological distress (GHQ)	25%	12%	37%	34%
Work engagement	4.01 (0.56)	4.07 (0.57)	3.64 (0.76)	3.61 (0.84)
Self-reflection	4.42 (1.10)	4.45 (0.98)	4.17 (1.00)	4.16 (1.05)
Compassion	6.08 (0.90)	6.11 (0.95)	5.96 (1.05)	6.02 (0.97)

### Tests of hypotheses

**Fig. 2:**
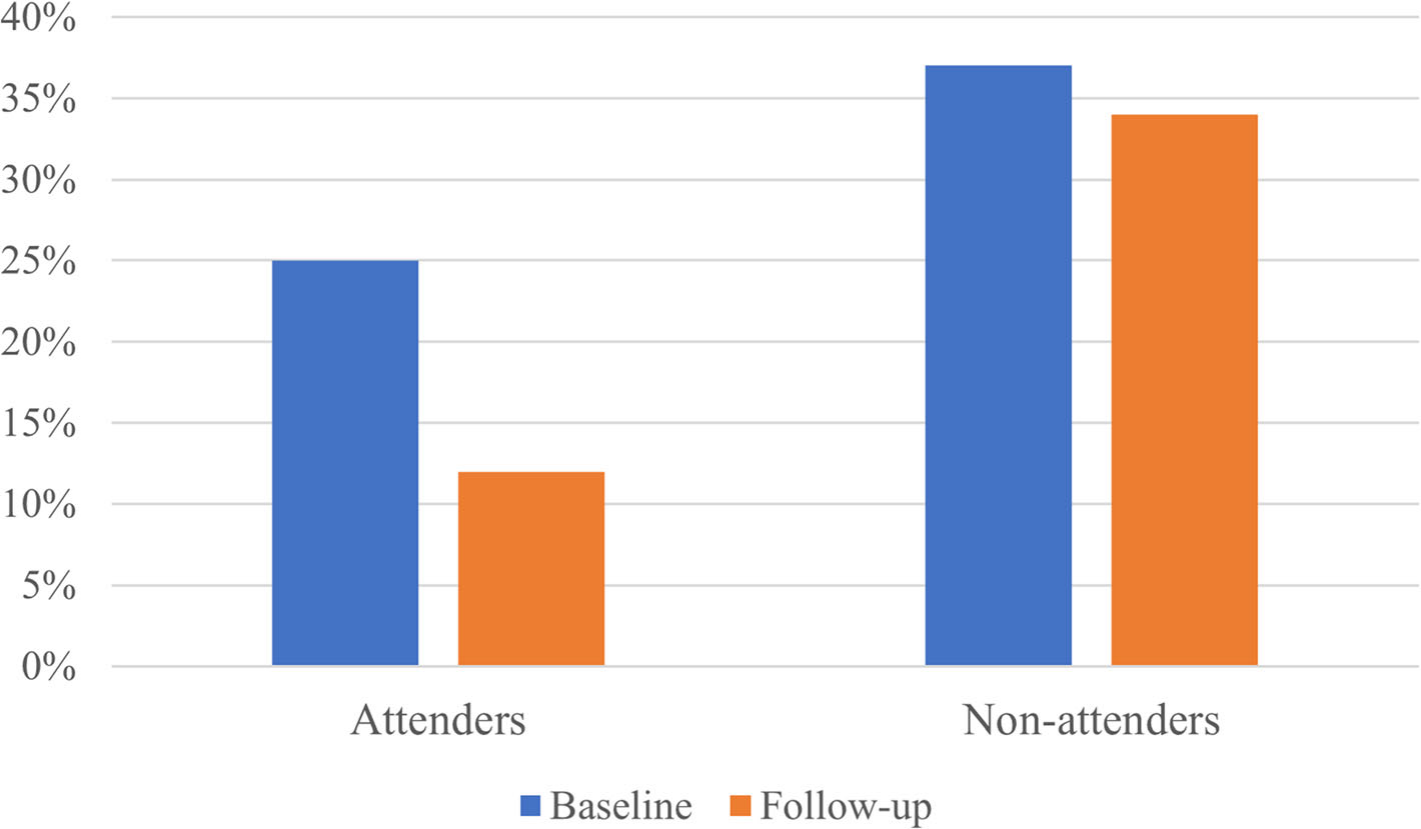
Change in Psychological Distress from Baseline to Follow-up for Regular attenders and Non-attenders

As shown in Table 2, there was a raw change in psychological distress in the intervention group from 25% at baseline to 12% at follow-up, compared with a much smaller drop from 37 to 34% in the control group. Figure 2 plots this data. However, this does not take into account the covariates, which were subsequently considered in the analysis shown in Table 3. This analysis revealed a significant effect of attendance (this effect converts to an odds ratio of 0.197; 95% confidence interval (0.047, 0.823)). That is, among attenders, there is a significantly greater decrease in GHQ scores (i.e. reduction in psychological distress) than among non-attenders. Expressed as an adjusted difference between the groups this is − 19%: that is, the decrease having taken all the covariates into account is even greater than the raw difference shown in Table 2. However, there was no significant effect of attendance on work engagement (B = 0.09; 95% confidence interval (− 0.16, 0.35)). There was no significant effect of attendance on compassion (B = 0.00; 95% confidence interval (− 0.29, 0.30)), or on self-reflection (B = 0.05; 95% confidence interval (− 0.32, 0.42)). Sensitivity analysis using Full Information Maximum Likelihood revealed the same pattern of findings.

**Table 3:** Results of regression analysis of outcomes on rounds attendance and covariates

	Outcome			
	Psychological distress	Engagement	Self-reflection	Compassion
Intercept	13.25 (− 655.13, 681.63)	1.84 (0.84, 2.84)**	1.19 (− 0.27, 2.65)	1.28 (− 0.07, 2.63)
Female	0.50 (− 0.36, 1.36)	0.06 (−0.16, 0.29)	−0.13 (− 0.44, 0.18)	− 0.08 (− 0.36, 0.19)
Nursing^a^	−12.60 (−680.98, 655.77)	−0.51 (− 1.39, 0.37)	0.15 (− 1.07, 1.37)	0.05 (− 0.95, 1.06)
Allied Health Professionals^a^	−12.43 (− 680.80, 655.95)	−0.60 (− 1.49, 0.28)	0.42 (− 0.82, 1.65)	0.07 (− 0.96, 1.09)
Other occupations^a^	−12.95 (− 681.33, 655.42)	−0.84 (− 1.75, 0.06)	0.29 (− 0.96, 1.55)	−0.26 (− 1.31, 0.79)
Part time	0.01 (− 0.92, 0.94)	− 0.07 (− 0.29, 0.16)	0.04 (− 0.28, 0.37)	0.03 (− 0.26, 0.31)
Grade: band 6^b^	−0.35 (− 1.26, 0.56)	0.01 (− 0.22, 0.23)	0.06 (− 0.25, 0.38)	0.18 (− 0.10, 0.46)
Grade: band 7^b^	−0.09 (− 1.12, 0.95)	0.00 (− 0.25, 0.26)	0.21 (− 0.15, 0.57)	0.12 (− 0.20, 0.43)
Grade: bands 8/9^b^	0.04 (−1.20, 1.27)	−0.04 (− 0.35, 0.28)	0.05 (− 0.41, 0.50)	0.35 (− 0.03, 0.74)
Grade: other^b^	−13.28 (− 681.66, 655.09)	−0.61 (− 1.61, 0.39)	0.37 (− 1.03, 1.77)	0.23 (− 0.92, 1.38)
Regular patient contact^c^	− 0.20 (− 1.29, 0.89)	−0.24 (− 0.52, 0.03)	0.16 (− 0.23, 0.54)	0.28 (− 0.13, 0.69)
Occasional patient contact^c^	−0.43 (− 1.77, 0.91)	−0.01 (− 0.36, 0.34)	0.20 (− 0.29, 0.69)	0.56 (0.02, 1.10)*
Age	0.09 (− 0.28, 0.46)	−0.01 (− 0.10, 0.09)	0.01 (− 0.12, 0.15)	0.14 (0.02, 0.25)*
Length of service	0.12 (− 0.11, 0.36)	−0.01 (− 0.06, 0.05)	−0.03 (− 0.11, 0.05)	−0.02 (− 0.09, 0.05)
Baseline value	−1.94 (− 2.68, − 1.21)**	0.72 (0.60, 0.83)**	0.65 (0.53, 0.77)**	0.67 (0.56, 0.78)**
Regular attender	−1.63 (−3.06, − 0.19)*	0.09 (− 0.16, 0.35)	0.05 (− 0.32, 0.42)	0.00 (− 0.29, 0.30)

Figures in main section of table are multilevel regression coefficients (95% confidence intervals), except for psychological distress, where the figures represent raw multilevel logistic regression coefficients

^a^ Reference group = medical/dental

^b^ Reference group = bands 1–5

^c^ Reference group = no patient contact

* *p* < .05 ** *p* < .01

## Discussion

This study evaluated the effectiveness of Rounds with healthcare staff from 10 UK healthcare organizations, all part of the NHS, except for one hospice. During the eight-month study duration, regular attenders participated on average four times (with Rounds typically held monthly). Our findings showed that Rounds significantly decreased the psychological distress of regular attenders compared to non-attenders; the adjusted odds ratio of 0.197 (95% confidence interval (.047, .823)), means that after taking background factors and baseline distress into account, the odds of experiencing psychological distress after attending Rounds regularly were 19.7% lower compared with non-attenders.. We however failed to find a significant effect of Rounds attendance on staff’s work engagement, compassion and self-reflection, meaning that we were not able to confirm the effectiveness of Rounds for these outcomes.

### Psychological distress

The finding that Rounds attendance was associated with a significant reduction in psychological distress is in line with previous research. Specifically, qualitative evidence and retrospective accounts pointed towards perceived negative effects of Rounds on work stress [19, 23]. These studies however only offer a weak/moderate evidence base for Rounds due to their weak study designs, such as lack of baseline measures or control group. Our findings thus advance these previous studies through rigorously evaluating Rounds with a pre-post control design to highlight that Rounds indeed contribute to healthcare staff well-being and mental health. In relation to the JD-R model [26], which informed our selection of outcomes, these findings support the argumentation along the health impairment route. Specifically, this route suggests that the observed changes in psychological distress following Rounds attendance stem from a reduction in job demands.

### Work engagement, compassion and self-reflection

Our inclusion of these outcomes was guided by the JD-R model [25] and previous research into Rounds [17–23]. While this research finds in its majority support that Rounds attendance positively contributes to work engagement, compassion and self-reflection and thus seemingly contradicts our non-significant findings, comparisons with our research are difficult to make. Specifically, our research aim was to address, through a sophisticated pre-post quasi-experimental design, the methodological limitations of this previous research (e.g., lack of baseline measure, measure of outcomes immediately after Rounds attendance) that hamper its ability to draw reliable conclusions regarding the effect of Rounds on work engagement, compassion and self-reflection. Given that our study represents a more conservative test, attendance at Rounds might indeed not lead to an increase in these outcomes.

Our selection of outcome variables had been theoretically informed by the JD-R model [26]. According to the motivational route of this model, changes in work engagement are brought about by changes in personal resources, such as compassion and self-reflection. Potential reasons for our lacking empirical support for the motivational route and inability to link Rounds attendance to these outcomes include differences between attenders and non-attenders. As opposed to randomized control trials (which may have been preferable as a test of the causal mechanism, but were not possible to be adopted because of ethical reasons and the inability to randomise due to the ad hoc and voluntary nature of attendance, and considerations regarding ecological validity), healthcare staff freely chose to attend Rounds, potentially resulting in self-selection bias. Our design represents a realistic portrayal of the implementation of Rounds in healthcare organizations [37], although Rounds might have attracted staff with certain interests (e.g., compassion in healthcare), who were currently struggling with problematic patient cases or who had the time to attend Rounds. These assumptions are supported by differences between attenders and non-attenders in baseline and demographic differences. Specifically, regular attenders already exhibited higher baseline levels of work engagement, self-reflection and compassion than non-attenders, while non-clinical and junior staff were less like to attend Rounds than other occupational groups. Although we controlled for the effect of these differences in our analysis, they might explain our inability to confirm changes in work engagement, self-reflection and compassion as recent changes in these outcomes might have motivated staff to attend Rounds, meaning that actual Rounds attendance resulted in little additional effect. This reasoning is also supported by the fact that our measure of psychological distress (GHQ), which revealed significant changes, constitutes a more objective indicator than the other outcome measures, that is less likely to be influenced by anticipated benefits of Rounds prior to attending. Another potential explanation is the relatively small number of regular attenders (*N* = 51), which might have affected our ability to detect effects [38]. While we had recruited a considerably higher number of participants at follow-up, a large number of these had to be excluded as they did not meet our inclusion criteria of attending 50% of Rounds offered by the respective healthcare organization. In the majority of cases, this was due to Rounds being implemented in complex multi-site organisations – rotating the location on a monthly basis - meaning that each Round was not necessarily accessible to individuals despite being available monthly at an organisational level. While we did not find quantitative evidence of differences in these outcomes, possibly for the reasons stated, it is worth noting that the wider study did find evidence of improvements in self-reflection and compassion in the qualitative realist evaluation [28], further supporting the notion that Rounds are beneficial in multiple ways.

## Conclusions

Our pre-post quasi-experimental evaluation based on data from 10 UK healthcare organizations offers a strong evidence base for the effectiveness of Rounds that overcomes serious limitations of previous evaluation studies (e.g., lack of baseline measures or a control group). In detail, our study revealed that Rounds attendance was linked to a 19% decrease in psychological distress (adjusted difference between attenders and non-attenders). As such, Rounds represents a particularly suitable intervention to assist healthcare staff in dealing with the ever-rising demands of the caring profession and promote their well-being and mental health. As staff health is closely linked to important patient outcomes, such as patient safety and mortality [8–10], Rounds should also be beneficial for recipients of care. Given that Rounds last on average only 1 hour, and aside from facilitator and admin time to prepare and host Rounds, require relatively low financial resources (i.e. provision of lunch and room and license fee), Rounds constitute a highly feasible and cost-effective way to decrease psychological distress and a potential avenue to improve healthcare staff’s well-being.

## Supplementary Information

**Additional file 1.**

## Data Availability

The datasets generated and/or analysed during the current study are not publicly available as some individuals may be identifiable given the characteristics included in the analysis. However, a partially anonymised version of the data is available from the corresponding author on reasonable request.

## Abbreviations

ANCOVA: Analysis of Covariance
GHQ: General Health Questionnaire
JD-R model: Job Demands-Resources Model
NHS: National Health Service
Rounds: Schwartz Rounds®
