# Supplementary material for: Effectiveness of a group intervention to reduce the psychological distress of healthcare staff: a pre-post quasi-experimental evaluation
Source: BMC Health Serv Res. 2021 Apr 27;21:392. doi: 10.1186/s12913-021-06413-4 (PMC8076663; doi:10.1186/s12913-021-06413-4)
Supplement: Supplementary file 1 — Additional file 1. [file 12913_2021_6413_MOESM1_ESM.pdf]

Organisation Code: A   Participant Code: __________

Survey of Staff Experiences

Longitudinal National Evaluation of Schwartz Center Rounds

Preliminary Question

Had you ever attended a Schwartz Center Round before the Round where you were given this questionnaire (including at a previous trust / employer)?	
☐ No	Please proceed to complete this survey.
☐ Yes	This survey is aimed at people who have not attended Schwartz Center Rounds.<br>As someone who has attended Schwartz Center Rounds before you are not eligible to participate in this survey. Please return this survey to the research team blank.

This is a questionnaire about your **views and experiences at work.**

This is **not a test**. There are no “right” or “wrong” answers. We are interested in your personal views. There is a short biographical section towards the end of the questionnaire. This is used to enable us to compare the views of different people across many organisations.

This questionnaire contains a unique ID number, to ensure your responses are anonymised, but so that we can match it up with the second questionnaire we will ask you to complete in 8 months’ time. All responses are confidential to the project team.

How do I fill in this questionnaire?

Please read each question carefully, and give your immediate response by ticking the box which best matches your views. We are interested in *your views*. Please answer all questions as openly and honestly as possible.

For example, a question in this survey is whether, ***in your opinion***, you have been able to enjoy your normal day-day activities. If you believe that this is the case, most of the time, you would tick the box “same as usual”.

Have you recently (over the past month)

	Better than usual	***Same as usual***	***Less so than usual***	***Much less than usual***
Been able to enjoy your normal day-to-day activities?	☐	☑	☐	☐

Consent to be contacted

☐ I confirm that I have read and understand the information sheet for this above study I understand that my participation is voluntary and that I am free to withdraw at any time. I agree to participate in the study by completing two questionnaires: one now, and one in eight months’ time. **Please tick the box on the left to indicate your consent.**

The following statements describe certain features and characteristics relating to your views, experiences and attitudes to work. Please tick the box that, in your view, most represents the situation that applies to you.

Section 1: About you

1. Have you recently (over the past month) *

a.	Been able to concentrate on whatever you're doing?	☐	*Better than usual*	☐	*Same as usual*	☐	*Less than usual*	☐	*Much less than usual*
b.	Lost much sleep over worry?	☐	*Not at all*	☐	*No more than usual*	☐	*Rather more than usual*	☐	*Much more than usual*
c.	Felt that you are playing a useful part in things?	☐	*More so than usual*	☐	*Same as usual*	☐	*Less useful than usual*	☐	*Much less useful*
d.	Felt capable of making decisions about things?	☐	*More so than usual*	☐	*Same as usual*	☐	*Less so than usual*	☐	*Much less capable*
e.	Felt constantly under strain?	☐	*Not at all*	☐	*No more than usual*	☐	*Rather more than usual*	☐	*Much more than usual*
f.	Felt you couldn't overcome your difficulties?	☐	*Not at all*	☐	*No more than usual*	☐	*Rather more than usual*	☐	*Much more than usual*
g.	Been able to enjoy your normal day-day activities?	☐	*More so than usual*	☐	*Same as usual*	☐	*Less so than usual*	☐	*Much less than usual*
h.	Been able to face up to your problems?	☐	*More so than usual*	☐	*Same as usual*	☐	*Less able than usual*	☐	*Much less able*
i.	Been feeling unhappy and depressed?	☐	*Not at all*	☐	*No more than usual*	☐	*Rather more than usual*	☐	*Much more than usual*
j.	Been losing confidence in yourself?	☐	*Not at all*	☐	*No more than usual*	☐	*Rather more than usual*	☐	*Much more than usual*
k.	Been thinking of yourself as a worthless person?	☐	*Not at all*	☐	*No more than usual*	☐	*Rather more than usual*	☐	*Much more than usual*
l.	Been feeling reasonably happy, all things considered?	☐	*More so than usual*	☐	*About same as usual*	☐	*Less so than usual*	☐	*Much less than usual*

**2.**	***For each of the statements below, how often do you feel this way about your job?***	*Never*	*Rarely*	*Sometimes*	*Often*	*Always*
a.	I look forward to going to work.	☐	☐	☐	☐	☐
b.	I am enthusiastic about my job.	☐	☐	☐	☐	☐
c.	Time passes quickly when I am working.	☐	☐	☐	☐	☐

* © David Goldberg, 1978

**3. To what extent do you agree or disagree with the following statements?**	*Disagree Strongly*	*Disagree*	*Disagree Slightly*	*Agree Slightly*	*Agree*	*Agree Strongly*
a. I don't often think about my thoughts.	☐	☐	☐	☐	☐	☐
b. I rarely spend time in self-reflection.	☐	☐	☐	☐	☐	☐
c. I frequently examine my feelings.	☐	☐	☐	☐	☐	☐
d. I don't really think about why I behave in the way that I do.	☐	☐	☐	☐	☐	☐
e. I frequently take time to reflect on my thoughts.	☐	☐	☐	☐	☐	☐
f. I often think about the way I feel about things.	☐	☐	☐	☐	☐	☐

Section 2: Interacting with patients

How often do you have contact with patients (*including: face-to-face contact, contact by telephone/email, etc.*)?

☐ Regularly (usually every day or every shift)   ☐ Occasionally (less often than this, but at least a few times a year)   ☐ Rarely/never

Please answer the following questions regarding your feelings about patients. If you do not have regular contact with patients, please answer the questions about other people you encounter at work, such as patients' relatives or your colleagues. If you are unable to answer any of the questions on this page, please leave those questions blank please go to **Section 3 (question 7)** on the next page.

**4. To what extent do you agree or disagree with the following statements?**	*Strongly disagree*	*Slightly disagree*	*Slightly agree*	*Strongly agree*
a. I am good at predicting how a patient will feel.	☐	☐	☐	☐
b. I am quick to spot when a patient is feeling awkward or uncomfortable.	☐	☐	☐	☐
c. I can sense if I am intruding, even if the patient does not tell me.	☐	☐	☐	☐
d. I can tune into how a patient feels rapidly and intuitively.	☐	☐	☐	☐
e. I can easily work out what a patient might want to talk about.	☐	☐	☐	☐

**5. How certain are you that you can successfully:**	*1 Not certain at all*	*2*	*3*	*4*	*5*	*6*	*7*	*8*	*9*	*10 Quite certain*
a. Initiate discussion with patients about their worries?	☐	☐	☐	☐	☐	☐	☐	☐	☐	☐
b. Encourage patients to talk about their feelings?	☐	☐	☐	☐	☐	☐	☐	☐	☐	☐
c. Uncover strong feelings such as anxiety and worrying?	☐	☐	☐	☐	☐	☐	☐	☐	☐	☐
d. End a conversation by summarizing problems and the agreed plan of action?	☐	☐	☐	☐	☐	☐	☐	☐	☐	☐
e. Pass on bad news to patients?	☐	☐	☐	☐	☐	☐	☐	☐	☐	☐

How certain are you that you can successfully:	1 Not certain at all	2	3	4	5	6	7	8	9	10 Quite certain
f. Confront patients in an appropriate way with something they are in denial about?	☐	☐	☐	☐	☐	☐	☐	☐	☐	☐
g. Appreciate that patients have a different understanding of the situation?	☐	☐	☐	☐	☐	☐	☐	☐	☐	☐
h. Help patients handle an uncertain situation?	☐	☐	☐	☐	☐	☐	☐	☐	☐	☐

6. To what extent are the following statements true of you?	1 Not at all true of me	2	3	4	5	6	7 Very true of me
a. When I hear about a patient going through a difficult time, I feel a great deal of compassion for him or her.	☐	☐	☐	☐	☐	☐	☐
b. I tend to feel compassion for patients, even when I do not know them well.	☐	☐	☐	☐	☐	☐	☐
c. One of the activities that provide me with the most meaning in my life is helping patients when they need help.	☐	☐	☐	☐	☐	☐	☐
d. I would rather engage in actions that help patients, than engage in actions that would help me.	☐	☐	☐	☐	☐	☐	☐
e. I often have compassionate feelings towards patients when they seem to be in need.	☐	☐	☐	☐	☐	☐	☐

Section 3:About your organisation

7. To what extent can you:	Not at all	To a small extent	Neither great nor small extent	To a great extent	Completely
a. Count on your colleagues to listen to you when you need to talk about problems at work?	☐	☐	☐	☐	☐
b. Count on your colleagues to back you up at work?	☐	☐	☐	☐	☐
c. Count on your colleagues to help you with a difficult task at work?	☐	☐	☐	☐	☐
d. Really count on your colleagues to help you in a crisis situation at work, even though they would have to go out of their way to do so?	☐	☐	☐	☐	☐

8. To what extent are the following statements true of you?	Definitely false	Mostly false	Mostly true	Definitely true
a. This organisation pays little attention to the interests of employees.	☐	☐	☐	☐
b. This organisation tries to look after its employees.	☐	☐	☐	☐
c. This organisation cares about its employees.	☐	☐	☐	☐
d. This organisation tries to be fair in its actions towards employees.	☐	☐	☐	☐

Biographical Details

This part of the questionnaire asks for details about you.
This information will be used to enable us to compare the views of different groups of people – it will not be used to identify you personally.

9. Demographic & background information

a. Age: ☐ 16-20 ☐ 21-30 ☐ 31-40 ☐ 41-50 ☐ 51-65 ☐ 66+

b. Gender:
☐ Male
☐ Female
☐ Male to female transgender
☐ Female to male transgender
☐ Not sure
☐ Prefer not to say
☐ Other (please specify): ______

c. What is your occupational group? (*Please tick one box only*)

Allied Health Professionals / Healthcare Scientists / Scientific and Technical

☐ Occupational Therapy
☐ Physiotherapy
☐ Radiography
☐ Pharmacy
☐ Clinical Psychology
☐ Other qualified Allied Health Professionals (*e.g. chiropody / podiatry, dietetics, orthoptics, arts therapy)*
☐ Other qualified Scientific and Technical or Healthcare Scientists (*e.g. haematology, clinical biochemistry, microbiology*)
☐ Support to Allied Health Professionals (*e.g. support worker, therapy helper, therapy assistant or student*)
☐ Support to Scientific and Technical or Healthcare Scientists *(e.g. technicians, assistants or students*)

Medical and Dental

☐ Medical / Dental – Consultant
☐ Medical / Dental – In Training (e.g. *Foundation Y1 & Y2, StRs (inc FTSTAs & LATs), SHOs, SpRs / SpTs / GPRs)*
☐ Medical / Dental – Other (*e.g. Staff and Associate Specialists / Non-consultant career grade*)

Registered Nurses

☐ Adult / General
☐ Children
☐ Midwives
☐ Other Registered Nurses (*e.g. mental health, learning disabilities, and district / community nurses, and health visitors)*

Nursing or Healthcare Assistants

☐ Nursing auxiliary / Nursing assistant / Healthcare assistant (*including Health / Clinical / Nursing Support Worker*)

Wider Healthcare Team

☐ Admin & Clerical (*including Medical Secretary*)
☐ Central Functions / Corporate Services (*e.g. HR, Finance, Information Systems, Information Technology*)
☐ Maintenance / Ancillary (*e.g. housekeeping, domestic staff, maintenance, facilities, estates)*

General Management

☐ General Management (*N.B. If you are a manager and can choose a group from elsewhere in the list, please select that other occupational group)*
☐ Directors and board members
☐ Commissioners
☐ Other general managers

Volunteers and non-employees

☐ Volunteer
☐ Not a member of this organisation

Other

☐ Other occupational group (*please specify*) ______

d. How many years have you worked for this organisation?
If your organisation has merged with another or changed its name, please include in your answer all the time you have worked with this organisation and its predecessors

☐ Less than 1 year ☐ 1-2 years ☐ 3-5 years
☐ 6-10 years ☐ 11-15 years ☐ More than 15 years

e. What is your grade?
c Not on an *Agenda for Change* contract c Band 1 c Band 2 c Band 3 c Band 4 c Band 5

c Band 6 c Band 7 c Band 8a c Band 8b c Band 8c c Band 8d c Band 9

f. Working hours
How many hours a week are you contracted to work?

☐ Up to 29 hours ☐ 30 or more hours

g. In total, on how many working days during the last 6 months have you been absent due to sickness? ☐

h. If you would like to tell us why you choose to attend Schwartz Center Rounds please do so here:

i. Did you complete this questionnaire before or after today's Schwartz Center Round? ☐ Before ☐ After ☐ Mixed / During

j. I would be happy to **take part in an interview** for the study and I am happy for a member of the research team to contact me to arrange this. ☐ Yes ☐ No

END OF QUESTIONNAIRE

Thank you for your time and effort in completing this questionnaire.
Please complete the questionnaire and hand it back to a member of the research team at the end of the Schwartz Center Round / or return by post in the pre-paid envelope provided. If you do not have an envelope please post back to Jeremy Dawson at the address below.

Longitudinal National Evaluation of Schwartz Center Rounds
Institute of Work Psychology, Sheffield University Management School,
Conduit Road, Sheffield S10 1FL
